# Adjustment to Chinese Culture and Mental Health Issues among Foreign Students on Chinese University Campuses during the COVID-19 Pandemic: A Collaborative Ethnographic Study

**DOI:** 10.3390/bs13070526

**Published:** 2023-06-22

**Authors:** Jian Li, Eryong Xue, Yunshu He

**Affiliations:** China Institute of Education Policy, Faculty of Education, Beijing Normal University, Beijing 100875, China

**Keywords:** cultural identity, international students, international higher education, higher education reform

## Abstract

Foreign students in China may have difficulty adjusting to Chinese culture and may experience mental health problems related to acculturation, interpersonal issues, and social communication within the context of campus life. Therefore, this study attempts to apply a collaborative ethnography approach to explore the adjustment to Chinese culture and mental health issues among foreign students on Chinese campuses during the COVID-19 pandemic. We spent 16 months exploring the feelings and perceptions of 82 foreign international undergraduate students at six Chinese higher education institutions regarding their adjustment to Chinese culture and gathered their suggestions about how to address the mental health issues experienced by foreign learners in China. The results show that international students tend to have a limited understanding of Chinese culture and rely on very few channels for information—in particular, the Internet, teachers’ lectures, and daily life—which can easily result in mental health problems and thoughts of marginalization. In addition, the results showed that international students’ mental health problems are subjectively positively correlated with their own personality, cultural intelligence, and cultural identification ability and objectively related to their cultural distance and all aspects of the educational work of international students. It is suggested that Chinese higher education institutions should strengthen their attention to the mental health of international students in China and promote international students’ cross-cultural adaptation abilities and understanding of Chinese culture.

## 1. Introduction

It is acknowledged that the greatest difficulty international students in China face is adjusting to Chinese culture, whereas they have only a medium level of difficulty dealing with acculturation, interpersonal and social communication issues, and school life during COVID-19 [1]. They also tend to adopt strategies that may marginalize them in society and daily life but are relatively more able to adjust to school rules, regulations, and behavioral norms [2].

Current studies concentrate on discussing cultural identity, its definition, content, characteristics, theoretical perspectives, and the adjustment to Chinese culture of international students in detail. Qin et al. (2022) argues that cultural identification refers to the psychological process of recognizing and imitating the attitudes and behaviors of others or other groups, making them part of one’s personality. Li (2015) propose that cultural identification involves the process whereby a person recognizes and agrees with the attitudes, customs, and behaviors of a specific external information source and internalizes them. In addition, Liu et al. (2022) argue that international students’ adjustment to a Chinese cultural context is a process that involves an individual’s conscious awareness of their individual cultural and social identity, which refers to their “awareness that he or she belongs to a particular social group and recognizes the emotions and meaning of being a member of a social group” through social classification [3]. Gu and Schweisfurth (2015) summarize the adjustment to Chinese culture as involving the sum of the material and spiritual productive capacity acquired and the material and spiritual wealth created by international students. International students’ adjustment to Chinese culture is considered to involve their spiritual productive capacity and spiritual products, including all forms of social consciousness, such as natural science, technical science, and social ideology. Considering the characteristics and theoretical perspectives on the adjustment to Chinese culture of international students, Li et al. (2015) insists that, as a historical phenomenon, Chinese culture is a historical inheritance. Cultures of different nationalities and regions form the diversity of human cultures. Yan and Berliner (2011) believe that a so-called “culture” is the “common psychological procedure” of the masses in the same environment. The cultural identity of international students is not an individual feature, but a common psychological phenomenon affecting groups with similar social experiences and a common educational background. Therefore, international students in different countries or regions naturally have different ways of thinking and cultural differences from their host countries due to their unique educational backgrounds and social, work, and life experiences [4].

There has been extensive discussion of mental health, its nature, definition, and characteristics in connection with international students during the COVID-19 pandemic. There is a close relationship between international students’ adjustment to Chinese culture and their mental health, which is the focus of this study [5]. For example, Li (2015) explores international students’ innovative strategies for managing their assimilation and directly addresses the relationship between cultural adjustment and mental health. Chen (2021) explains that, for international students in China in the context of the COVID-19 pandemic (and the post-COVID-19 period), the greater their difficulty with acculturation and adjustment to Chinese culture, the more serious their mental health symptoms. In addition, Li et al. (2010) point out that there is currently a mismatch between China’s comprehensive national strength and its image in the international community. Li and Xue (2022a) highlight the following aspects as mainly reflecting the psychological impact of the pandemic on overseas students: loneliness and boredom, anger caused by dissatisfaction with the government’s response to the pandemic, and fear of discrimination among infected patients and their families. For instance, teaching methods have also changed during the epidemic—from classroom to online teaching—and many international students must swap night and day due to time differences. This will put serious psychological pressure on international students. Dealing with life in a foreign country, building a social circle from scratch, facing the dual pressures of dealing with schoolwork and looking for future employment, and coping with culture shock all contribute to international students being considered “at risk” for depression [5].

### 1.1. Cultural Identity and International Students

Before exploring the adjustment to Chinese culture and mental health issues among foreign students on Chinese campuses during the COVID-19 pandemic, it is worth reviewing how different scholars have defined cultural identity. For example, Gu and Schweisfurth (2015) argued that cultural identity is the consensus and recognition of human beings toward culture [6]. International students’ cultural identity is considered the social psychological process through which these individuals internalize and belong to a culture and a cultural group to obtain, maintain, and innovate their own culture—it is a process of self-formation. According to Maeder-Qian (2018), in social life, cultural identity is always associated with specific cultural patterns, showing the degree of recognition of a common culture among its members. International students’ cultural identity can be understood as the processes through which these individuals and groups define themselves, strengthen each other’s sense of identity, and condense into groups with common cultural connotations. With the ongoing intensification of globalization and the integration of global cultures, the number of multilingual learners is also increasing, among whom cultural identity usually extends to the confirmation of self-image and common culture. It can be regarded as mutual familiarity, acceptance, and internalization between different cultures to finally practice [7].

For international students, negotiating issues of cultural identity involves the acceptance and recognition of different cultural characteristics, including cognition, emotion, and behavior. For example, Ratnapalan and Haldane (2022) argued that a sense of shared cultural identity among international students is mainly manifested by sharing a common cultural background and cultural atmosphere or by recognition and acceptance of each other’s cultures. It contains both a part of an individual’s culture and a part of the other’s culture. When facing other cultures, people need to adjust their cultural needs and responses, make sure to preserve the harmony between themselves and the social and cultural environment in which they find themselves, seek a foothold in their own culture, and explore the significance of their own culture through conflict and collision with different cultures [8].

### 1.2. General Context of International Students’ Cultural Adjustment and Mental Health during the Pandemic

The recent studies also investigated the cultural adjustment and mental health status of international students during the epidemic period and its influencing factors. For example, Xu et al. (2022) adopted an emergent public health events questionnaire, emotion regulation strategy questionnaire, and social support questionnaire to investigate the influence of social support and emotion regulation on the mental health of international students [9]. The results showed that during the COVID-19 epidemic, the international students showed mild psychological symptoms overall, which was healthier than a similar investigation during the SARS period. In addition, Bourgeois and Zare (2023) also showed that social support was significantly negatively correlated with psychological problems, social support was significantly positively correlated with both emotion regulation strategies, expression inhibition strategies were only negatively correlated with fear and hypochondriasis, and emotion regulation strategies had a partial mediating effect between social support and fear symptoms. In conclusion, social support can not only directly reduce the risk of psychological problems for international students in China but also indirectly reduce psychological problems by influencing the use of emotional regulation strategies. During the epidemic, depression stress reaction will aggravate patients’ emotional problems, such as irritability and anxiety, and even lead to adverse effects, such as physical problems. Kruze et al. (2021) have also shown that the overall emotional level of international students in China is not good during the major epidemic situation. For a long time, the management of domestic universities for international students has mostly focused on creating better material conditions and cultural environment for them, but paid insufficient attention to the spiritual needs of international students. Taniguchi et al. (2022) also found that severe epidemic prevention and control, language communication barriers, and academic pressure will increase the negative emotions of international students. In addition, the self-care ability of foreign students is stronger than that of local students, which is mainly related to the relaxed education style that foreign students have received since childhood, so they are more tolerant to failures and shortcomings, rather than denying everything about themselves. Bhat et al. (2021) highlighted that self-caring ability is significantly positively correlated with positive emotions and negatively correlated with negative emotions during the pandemic period. In general, during the epidemic period, international students’ emotions, mental health, or sense of identity would be problematic to a certain extent [10,11,12].

### 1.3. Theoretical Perspectives on Cultural Identity

Most theories of cultural identity have been developed in the contexts of identity theory and cultural theory. Theories related to identity have been studied in many disciplines, such as philosophy, sociology, psychology, education, and communication. Identity theory is essentially a set of ideas about identity and cultural identity. Social identity theory is an important theory for revealing group behavior and intergroup relations [13]. It was proposed by British social psychologist Tayver. This theory mainly emphasizes that individuals recognize that they belong to a specific social group and that, at the same time, they will be aware of the emotional and value-based significance associated with being a member of the group. If individuals belong to a group, they will obtain a kind of social identity and share a kind of group representation. Intergroup attitudes and behaviors reflect the objective interests between one group and other groups, as well as many other ideas. Individual self-identity is often related to individual psychology. For developmental psychology, the process of development of an individual’s cultural identity is one in which, as an individual grows up, their level of cultural identity will keep increasing until a stable sense of national identity is formed at a certain age. Individuals’ social identities promote the construction of their self-identity [14,15,16]. During the process of communication, individuals both internalize social interactions into their identity and verify their existing identity through social interactions. From a psychological perspective, self-verification theory holds that individuals are more prone to depression when others’ evaluations of them are inconsistent with their own self-concepts [17]. According to the Silencing the Self-theory, when an individual engages in inauthentic self-expression—for example, by showing a non-real self or suppressing the performance of the real self—this will lead to self-division within the individual and inner chaos, and thus lead to depression. This can be explained by the self-discrepancy theory. Self-difference leads to self-inconsistency, which can easily cause emotional problems that can affect mental health and even result in serious mental illnesses [18].

Some scholars have pointed out that social identity is a collective form of value identity, group identity, self-identity, and cultural identity. In the process of social communication and social identity, there must also be a process of cultural identity. Stuart Hall’s theory of cultural identity was put forward in the post-colonial era, with ethnic diaspora as the fulcrum. Cultural identity is the search for history, which condenses a nation into a cultural group through culture. It is the identification of a “common culture” that reflects a nation’s identification with shared historical experiences and cultural norms. In the process of globalization, population flows have become more frequent and larger, and the flows and integrations between cultures have increased correspondingly [19]. The American anthropologist Robert Redfield and his colleagues proposed that acculturation is the phenomenon, whereby two cultural patterns change during the continuous interaction between individuals or groups with different cultural backgrounds after they enter a new social environment. The learning process in cross-cultural adaptation theory holds that the process of cross-cultural adaptation is a process of continuous learning. Various scholars have described the different stages of the process of acculturation. Acculturation is a dynamic process, including the beginning of the honeymoon period, the withdrawal of novelty, and the return to a higher level of acculturation. In cross-cultural communication, an individual or group culture learns from another culture and adjusts to develop the adaptation process, which is a long-term process of accumulation manifested as a dynamic form of pressure–adjustment–progress [20]. This process is like a spiral spring, moving two steps forward and one step back, gradually advancing under pressure. According to this theory, a person who moves from their inherent cultural environment to a new one will experience cultural maladaptation and find that their commonly used symbols of social communication cannot be used. The resulting sense of anxiety and loss will lead to hostility and a negative evaluation of the new environment and culture, as well as irrational attachment to the original cultural environment. Individuals in the new cultural environment act, to a certain degree, as “cultural receivers”, while cultural disseminators try to make efforts to promote their own culture and encourage new individuals from different countries to identify with it. Burke’s identity theory emphasizes that communicators can build a sense of identity with an audience by emphasizing common or similar characteristics, emotions, thoughts, and values. In terms of content rhetoric, the communicator can achieve “identity” with the target audience by adopting three strategies: opposition identification, sympathy identification, and error identification [21,22,23,24].

### 1.4. Chinese Culture and International Students

Promoting an appreciation of Chinese culture among international students in China is a matter of great significance. It involves promoting the studies and lives of international students in China, facilitating the management of universities, promoting national image communication, and promoting the national culture through soft power. Through empirical investigation, some scholars have analyzed shortcomings in terms of how current international students relate to Chinese culture—for example, by having little or only a shallow understanding of Chinese culture, excessive reliance on single channels of information, and weak subjective willingness—and have put forward corresponding countermeasures and suggestions [25]. Suggestions have also been put forward on how to cultivate international students’ sense of appreciation for Chinese culture from the perspectives of national policies, administrators, teachers, and service personnel team construction, teaching, and network platforms [26]. Promoting an appreciation of Chinese culture is a very important part of international students’ education, and cultural education is certainly an indispensable part of this. The cultural conflicts and differences in the educational policies of Chinese students in China have been explored from the perspectives of various cultural dimensions, from political aid to construction, reform, and opening to deepening reform [27].

As the focus of education for international students in China has shifted from respecting cultural differences to promoting an appreciation of Chinese culture, the Ministry of Education has repeatedly emphasized the importance of assimilation management, but there are still some problems in practice. An appreciation of Chinese culture should be promoted to ease cultural conflicts, accelerate the efficient growth of convergent management, and put forward corresponding innovative strategies, mainly with regard to the management system and its mechanisms. Some scholars have explored the innovation path of convergent education from the perspective of culture and identity [28]. Convergent education should pay attention to the essential construction of the education field, emphasizing the human role within culture, and make it clear that students coming to China need to recognize both their rights and their obligations in China’s education system [29].

### 1.5. Adjustment to Chinese Culture and International Students’ Mental Health

The study of cross-cultural adaptation pays more attention to cultural identity at the individual level. When international students fail to deal properly with the process of acculturation—such as by making an insufficient effort in language learning or social interaction or by internalizing negative stereotypes about Chinese culture—they become prone to anxiety, depression, and other mental health problems. As mentioned earlier, the greatest difficulty international students in China face is adjusting to Chinese culture, whereas they have only a medium level of difficulty dealing with acculturation, interpersonal and social communication issues, and school life. Moreover, they tend to adopt strategies that may marginalize them in society and daily life but are relatively more able to adjust to school rules, regulations, and behavioral norms [30]. At the same time, the greater the difficulty students have with acculturation and adjustment to Chinese culture, the more serious their mental health symptoms. Cultural intelligence has a significant positive impact on intercultural identity integration, and cross-cultural anxiety plays a mediating role between the two, while cultural distance also affects the mediating effect of cross-cultural anxiety. In other words, cultural intelligence, cultural distance, and cross-cultural anxiety all affect the integration of bicultural identity. In addition, cross-cultural adaptation inevitably puts forward requirements for students’ cross-cultural communication abilities. When international students’ cross-cultural communication ability is insufficient, it can easily affect their social lives, thus leading to mental health problems. Individual-performance identification differences and individual-relationship identification differences have been found to be positively correlated with depression [31].

At the same time, a deficit in cross-cultural communication ability does not lead directly to depression, but through the individual mediation of individual-performance identification difference and the chain mediating effect of individual-performance identification difference and individual-relationship identification difference. It is acknowledged that promoting international students’ appreciation of Chinese culture is of great strategic significance to the international presentation of Chinese stories. For example, it can help international students become friendly ambassadors of Sino-foreign cultural exchanges, firm defenders of China’s image, and international narrators of Chinese stories [32]. At the same time, corresponding strategic measures have also been put forward from the perspectives of strategic awareness, objectives, curricula, educational methods, and management. In addition, some scholars have discussed the significance, strategies, and paths of international students in the external communication of a certain kind of specific culture. The first issue is how to tell Chinese stories well to international students, and the important reason for telling Chinese stories well to international students is to promote their positive perception and understanding of Chinese culture, promote the transmission of Chinese values, and show China’s international image. Meanwhile, some scholars have taken traditional Chinese medicine culture as an example to discuss the positive mediating role of Chinese culture in media contact and cross-cultural communication [33,34,35,36].

### 1.6. Cross-Cultural Adaptation of Culture by Foreign Students

In terms of learning, many scholars tend to study the sense of belonging brought by international students in terms of social integration and related aspects, but the sense of belonging in academic and professional aspects is also very important. Many international undergraduate students studying engineering in the United States are more inclined to stay in the academic world for further development. Therefore, colleges and universities should provide students with academic development support and pay attention to non-academic support [37]. Academic writing ability is very important for graduate students. Most academic writing courses are limited to reading and writing, and some scholars have proposed that peer discussions with individuals from multicultural backgrounds can broaden perspectives and thinking in academic writing [38]. At the same time, teachers should give proper guidance to students’ discussions rather than leaving it to them to discuss freely. Such a curricular model for academic writing not only helps students develop perspectives on their theses but also helps teachers diversify their curriculum designs. Some scholars believe that listening to the opinions of experts with multicultural backgrounds will also help open new perspectives on writing for international students.

Since the pandemic, students for whom it was not convenient to go abroad have also been able to participate in online learning. While online courses bring some convenience, various forms of interactive, autonomous, or framed teaching or learning modes, as well as cross-cultural, international, and diversified online course systems, are needed to improve cross-cultural communication in online learning. Whether in online or offline teaching, we should pay attention to the application of the group teaching methods. Some scholars believe that in international student groups, cultural intelligence is helpful for reaching a consensus within the group and improving the efficiency of cross-cultural groups. At the same time, intra-group identity plays an intermediary role between cultural intelligence and cross-cultural group efficiency [39]. The collectivism of Asian cultural values makes Asian students more likely to seek help from social organizations and friends than from specialized mental health services. In addition, those with a higher sense of discrimination and a lower sense of Asian cultural values show a higher demand for psychological services, while those with a higher sense of Asian cultural values tend to have a lower demand for mental health services. Moreover, some students are more likely to abandon aspects of Asian culture that conflict with mainstream culture after experiencing discrimination. Therefore, it has been suggested that colleges and universities should make psychological counseling services more available to international students, for example, through informational sessions before the beginning of the semester to introduce school and community psychological counseling services. In addition, psychological service providers should fully consider the cross-cultural nature of Asian students. It is suggested that more consideration should be given to the views of international students who are discriminated against in the campus environment, and that more information should be collected so that schools and teachers can provide more appropriate services to students. In terms of work, some scholars have explored the advantages and disadvantages of overseas students’ study experiences for job hunting. The advantages and the disadvantages are some cultural conflicts [40].

However, the current academic research tends to focus on issues of cultural identity, and there have been relatively few studies on how international students deal with adjustment to Chinese culture, especially during the COVID-19 pandemic. Thus, the purpose of this study is to apply a collaborative ethnography approach to explore the adjustment to Chinese culture and the mental health of foreign students on Chinese campuses during the COVID-19 pandemic. This study concentrates on clarifying the relevant definitions and theories of cultural identity, as exploring how international students deal with the process of adjustment to a Chinese cultural context will help propose appropriate countermeasures to improve the mental health of international students and offer directions for future research.

The research questions are as follows:

Q1: What difficulties do international undergraduate students in China face with respect to their adjustment to Chinese culture and mental health?
Q2: What are the feelings, perceptions, and attitudes of international undergraduate students in China regarding their adjustment to Chinese culture and mental health issues?

## 2. Materials and Methods

### 2.1. The Collaborative Autoethnography Approach

We applied collaborative ethnography as a qualitative research method to provide an in-depth understanding of the sociocultural experiences of selected international students in China. The idea of collaborative autoethnography is considered a key part of autoethnography, and it concentrates on integrating both sociocultural analysis and interpretation with a narrative approach. It aims to address the specific problems and difficulties of conducting autoethnography by deleting various perspectives to balance individual narratives. The collaborative autoethnography approach is regarded as a key method for offering a specific lens on the experiences of multidisciplinary groups when conducting research amid complexity and intersectionality. Ethnography is a field study method derived from anthropology in which participants participate, publicly or privately, in the lives of a particular group of people over a long period of time, providing an internal picture of meaning and behavior. Ethnography uses the individual researcher as a research tool to depict a social group in detail and accurately by collecting a wide range of materials and paying attention to people’s common and habitual activities [41].

### 2.2. Research Procedures and Processes

In this study, following the research procedures for collaborative autoethnography, we generated six stages that we planned to undertake. In the first stage, we started to identify and explore the research topic of mutual interest to the researchers involved and to determine each of their roles and major duties in the study. In the second stage, we followed the research questions to examine the key themes regarding international students’ adjustment to Chinese culture. Key stakeholder engagement has been included in our study and is based on the proposed key stakeholders involved in international students’ adjustment to Chinese culture. In the meantime, we also discussed the uncertainties faced by international students in China, especially during the post-COVID-19 period. The researchers comprehensively explored the challenges and difficulties associated with adopting this approach. In the third stage, following the identified key themes, we conducted the research and divided the key themes into specific research topics. Then, we integrated the individual reflections and, based on them, conducted the thematic analysis accordingly. In this stage, after conducting the interviews, based on previous relevant studies and analysis of the current interview transcripts with a view to exploring issues related to the adjustment to Chinese culture and mental health issues among foreign students on Chinese campuses during the COVID-19 pandemic, we conducted a screening for key themes that followed two principles: one looked for high-frequency words, and the other identified the key themes of discussion in key sentences, including summarizing sentences, looking for the viewpoints of sentences, and assessing the attitudinal tendencies of sentences. For analysis of this type, the NVivo software is intended as a way of collecting a wide range of materials and gathering information about common activities.

**Figure 1:**
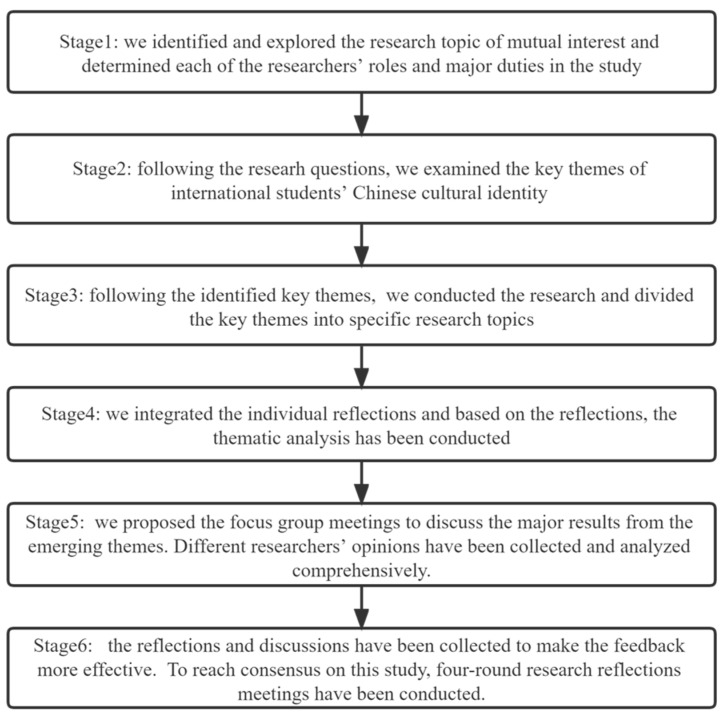
The research framework and procedures.

In the fifth stage, we proposed focus group meetings to discuss the major results from the emerging themes. After the transcription of the interview content is completed, the researchers need to identify the key themes and important information in the interview transcript relevant to the research topic, summarize it into a single list or condense it into short word lists, and then combine the findings with theoretical achievements in related disciplines in terms of human society’s basic behavioral habits and other factors for in-depth analysis. Different researchers’ opinions should then be collected and analyzed comprehensively. In the sixth stage, the reflections and discussions should be collected to make the feedback more effective. To reach a consensus on this study, four roundtable research reflection meetings were conducted. In addition, we used both transcripts and summaries to present the participants’ expression in the result (See Figure 1).

### 2.3. Ethical Considerations

In collaborative autoethnography, ethical consideration involves ensuring that participation is entirely voluntary, the focus is mutually agreed upon, and the collaboration is non-hierarchical and non-coercive. For trust and solidarity, we attempted to maintain friendships with the participants. All the researchers clearly defined their research roles and responsibilities to maintain a friendly working relationship and make a smooth progress of our study. Our researchers maintained close connections with the staff, teachers, and students of the selected higher education institutions. During the COVID-19 pandemic, we also encountered some challenges in collecting data, and we could no longer come together to meet in person. We chose to conduct collaborative autoethnography analysis through online Tencent meetings as our preferred option. For trust and solidarity, we maintained sustained engagement with the stakeholders at the selected higher education institutions to increase the quality and validity of the data collection process.

### 2.4. Descriptive Data Analysis of the Participants

A total of 82 international students participated in this study (20 students from the US, 16 from the UK, 18 from Korea, 10 from Japan, 9 from Canada, and 9 from New Zealand). There were 56 female participants and 24 males. Among them, there were 9 students in Class 16 of the undergraduate mathematics major (Grade 21); there were 2 students in Class 13, 2 students in Class 1, and 1 student in Class 4 of the undergraduate English major (Grade 20). There was 1 student in Class 2 of the undergraduate mathematics major (Grade 20); there were 5 students in Class 1 of the undergraduate geography major (Grade 21); and there were 3 students in Class 4, 6 students in Class 5, and 3 students in Class 6 of the music major (Grade 21). There were 7 students in Class 1 and 5 students in Class 6 of the undergraduate accounting major (Grade 21). There were 5 students in Class 4 and 1 student in Class 8 of the undergraduate art major (Grade 21). There were 17 students in Class 1 and 8 students in Class 2 of the digital media major; there were 3 students in Class 2 of the undergraduate psychology major (Grade 21); and there were 4 students in Class 4 of the undergraduate primary education major (Grade 21). In general, from the perspective of grades, there were 79 undergraduates in Grade 21 and 3 undergraduates in Grade 20. In terms of majors, there were 9 students majoring in Chinese, 5 students majoring in English, 1 student majoring in mathematics, 5 students majoring in geography, 12 students majoring in music, 12 students majoring in accounting, and 6 students majoring in fine arts. The participants were located in Beijing, Shanghai, Guangzhou, Hangzhou, and Tianjin and ranged in age from 18 to 26 years. We used convenience sampling to recruit participants, meaning that the researchers chose people they encountered by chance as survey objects for the convenience of carrying out their work according to the actual situation, or just selected those who were closest and easiest to find as survey objects. The primary researcher is a faculty member specializing in the study of international students at colleges and universities. He has encountered many international students from different countries through his administrative work in the international departments of colleges and universities. Thanks to introductions and recommendations from overseas students, he was able to interview more overseas students from other universities and majors (see Table 1).

## 3. Results

Three researchers and six research assistants were engaged in the whole collaborative ethnography research process from May 2021 to October 2022. We engaged in both online (through WeChat and the Tencent Meeting software) and face-to-face (in the class, library, and dormitory) interactions with selected participants. We confirmed that the greatest difficulty international students in China face is adjusting to Chinese culture, whereas they have only a medium level of difficulty dealing with acculturation, interpersonal and social communication issues, and school life and that they tend to adopt strategies that may marginalize them in society and daily life but are relatively more able to adjust to school rules, regulations, and behavioral norms. With the increasing number of international students in China, the problems they are facing are becoming increasingly prominent, and the most important problem is adjusting to Chinese culture. Due to the heterogeneity of international students, the diversity of cultural backgrounds, and the different degrees of cultural distance between their home cultures and Chinese cultures, there were various multi-level and distinct problems in terms of their adjustment to Chinese culture.

### 3.1. Internal Difficulties of International Undergraduate Students with Respect to Adjustment to Chinese Culture and Mental Health

For international students, cultural identity involves both the group culture and how individuals are affected by the group culture. Cultural identity is people’s tendency, consensus, and recognition of a certain culture, as well as people’s attribution consciousness to culture.

Focusing on the perspective of cultural cultivation and education, some scholars have analyzed the practical challenges, value criteria, and education paths faced by Chinese colleges and universities in providing cultural education to international students in China from an intercultural perspective. Interculturality advocates diversity, equality, respect, understanding, and interactive dialog in terms of aspects of the educational concept, campus environment, teaching method, and so on. It proposes a logical approach to the education of international students in China from the four perspectives, i.e., goals, values, cultures, and practices. In terms of the cultural dimension, promoting the adjustment to Chinese culture of international students in China can promote unity between the two peoples, consolidate public opinion, and provide a strong foundation for the construction of a community with a shared future for mankind.

**Figure 2:**
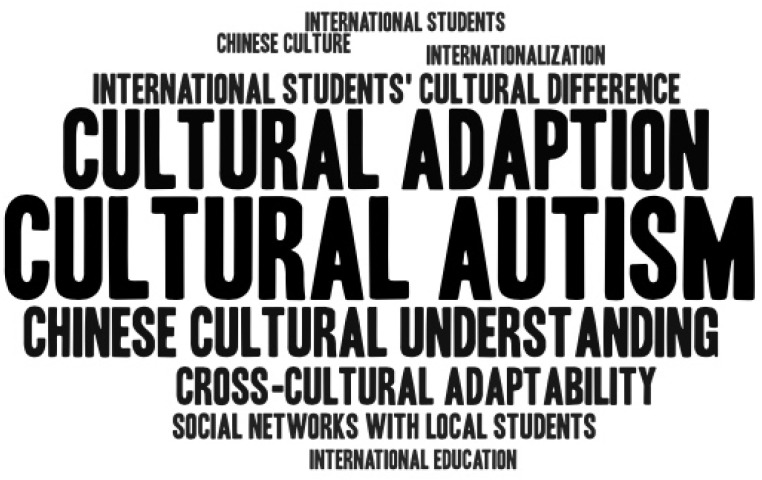
The cloud word analysis of top 10 high-frequency words.

The qualitative analysis for coding was conducted using the NVivo 11 software to collate the collected interviews about the soft power transformation of international students, and a series of text analysis results were obtained. NVivo is a qualitative analysis software package that supports both qualitative and hybrid search methods. The software can collate and analyze unstructured or qualitative data. We took the individual interview cases as a whole to conduct the high-frequency word analysis and set the word length to at least 3 to avoid invalid words or function words. Here, we define high frequency as content words with a weighted percentage of more than 0.1%—that is, as words that appear at least once in 70% of the interviews. By comparing the high-frequency words in each interview, we found the top 10 highest-frequency words in the results, i.e., “Cultural autism,” “Cultural adaptation,” “Chinese cultural understanding,” “Cross-cultural adaptability,” “International students’ cultural difference,” “Social networks with local students,” “Chinese culture,” “International education,” “Internationalization,” and “International students.” Based on the results of the coding analysis, we found that “Cultural autism” and “Cultural adaptation” were the two highest-frequency words in the interview transcripts (see Table 1 and Table 2 and Figure 2).

International students’ cognition of Chinese culture affects their adjustment to Chinese culture. At present, international students still have a limited understanding of Chinese culture and tend to rely on very few channels for information—for example, the Internet, teachers’ lectures, and daily life—which can easily result in their holding subjective and one-sided feelings about China. Some students are unwilling to understand the essence of Chinese culture, and their understanding is superficial. They tend to ignore the core values of Chinese culture in their lives and studies. One participant highlighted that:

*From my own experience, I always feel like I am suffering from cultural autism. I think the cross-cultural adaptability of international students is important. I am more likely to have weak cross-cultural adaptability due to my [home culture’s] cultural distance [from Chinese culture], introversion, lack of Chinese language proficiency, and weak cultural integration ability. As a result, I am unable to integrate into study, daily life, or the community in China, and I even tend to have psychological and mental problems, such as depression, anxiety, and so on, which further hinder my adjustment to Chinese culture.*

(WYX)

Understanding Chinese culture is the primary problem. When international students have a good understanding of China, their cross-cultural adaptability will be enhanced, which will further promote their adjustment to Chinese culture. At present, international students’ adjustment to Chinese culture is insufficiently understood in domestic academia, as research has mainly been conducted through empirical questionnaires, and the conclusions remain at the level of statements of fact. The education of international students is also closely related to their adjustment to Chinese culture. International students’ understanding of, and adjustment to, Chinese culture is a long-term process of psychological construction that needs to be understood in terms of the psychological level, practical level, and other aspects. As foreign students have their own cultural backgrounds, we need to deal with the relationship between the foreign students’ cultures and Chinese culture in a proper way. Emphasizing the cultural confidence of relevant staff and promoting international students’ adjustment to Chinese culture are exactly our principles in dealing with different cultures. This is an important way of improving the quality of management of international students and an effective way to cultivate international graduate students. At the psychological level, colleges, universities, and administrators should first be confident in their own understanding of Chinese culture, adopt a people-oriented approach to the management of international students, and correctly handle the relationship between international students’ cultures and Chinese culture. Only when universities and administrators truly understand and practice cultural confidence, they can encourage overseas students to trust Chinese culture. In practice, colleges and universities should look for various ways to provide international students with as many practical opportunities to get in touch with, understand, and practice Chinese culture as possible to enhance their favorable impression and recognition of Chinese culture. The psychological and practical levels complement and promote each other. When Chinese cultural identity becomes the primary task for international students, the education of international students also exposes corresponding problems that are not conducive to promoting the adjustment to Chinese culture of international students. One participant argued:

*During the COVID-19 pandemic, we had to take online courses and had no opportunities to meet other students. We felt lonely and felt the need to communicate with teachers, parents, and friends. From my observations, in terms of school management, universities should consider diplomatic and political factors and give special management and “differential treatment” to international students in life and study to foster their adjustment to Chinese culture and improve the quality of education. In recent years, the Ministry of Education has also vigorously advocated “convergence management,” but some schools still engage in formalism and other deviations in the process of implementation. In terms of course content, for cultural courses, Chinese culture has not been clearly written into the course syllabus, and traditional culture has been overemphasized, while modern innovative achievements have been ignored.*

(ZXL)

### 3.2. The External Difficulties of International Undergraduate Students: Professional Courses and Campus Environment

For professional courses, cultural heritage and the penetration of Chinese core values are easily ignored. In terms of the teaching methods and models used in international students’ education, they are more dependent on the classroom settings and textbook content, which results in a lack of practical interaction in the second classroom. Improving the relevant courses for international students is an important guarantee for facilitating their adjustment to Chinese culture. Promoting curriculum reform for international students and integrating Chinese traditional culture into the curriculum for international students can not only realize the combination of professional knowledge courses and humanistic courses but also set the scene effectively for the spread of traditional Chinese culture in the form of courses on relevant topics and broaden the audience for traditional Chinese culture. This can improve the effectiveness of Chinese cultural communication and provide a guarantee for the realization of overseas students’ adjustment to Chinese culture. In the face of international students with multicultural backgrounds, teachers and other staff members also have the problem of insufficient cross-cultural working ability. One participant argued:

*I found that the campus environment of the school still has its own characteristics—it is excellent at reflecting aspects of Chinese culture, but its reflection of multiple cultures is not obvious. In addition, the communication channels between international students and local Chinese students are insufficient, so international students seldom interact with Chinese students, which is not conducive to narrowing cultural distance and increasing adjustment to Chinese culture.*

(JYF)

The external environment and education are both important factors that affect people’s growth and development, and the education of international students is also the education of people. Therefore, it is necessary to think about the promotion of the adjustment to Chinese culture of international students from a multi-level perspective of the most original education. Therefore, it is suggested that schools should strengthen the cultivation of the intercultural communication abilities of international students. Higher education institutions should strengthen their attention to the mental health of international students in China and promote international students’ cross-cultural adaptation and adjustment to Chinese culture. One participant further argued:

*In my opinion, one of the most important strategic significances of the education of international students in China is that they can help tell Chinese stories and spread Chinese images through the important medium of international students in China. Therefore, there are many scholars specializing in the study of the significance of Chinese culture for international students in the communication of national images and of measures to improve their understanding of Chinese culture. In the context of telling Chinese stories well on the international stage, the analysis suggests that regarding the important communication channels and realization paths for international students to tell Chinese stories well as “others,” it is necessary to pay attention to the education of international students in Chinese culture, enhance their adjustment to Chinese culture, and fully accumulate their Chinese story content and mirror materials so as to better speak through the voice and convey the voice through the tube.*

(ZCY)

### 3.3. International Undergraduate Students’ Implicit Difficulties: Intercultural Competence and Cross-Cultural Adaptation

During the analysis of our collaborative autoethnography, we found that most of the participants realized that intercultural competence is one of the most important abilities for the 21st century and life-long learning. One participant pointed out:

*During my period of learning Chinese, I felt frustrated and found that there are many factors that affect cross-cultural competence and that these factors will also affect cross-cultural adjustment, cultural integration, social adaptation, and so on. These factors include, for example, the relationships among intercultural strategies, ethnic identity, cultural distance, and the sociocultural adaptation of overseas students, and the results show that cultural distance has a certain impact on sociocultural adaptation. It is not easy to live and study in a foreign country, and we need to learn a lot about cultural differences and identities.*

(HXD)

We found dynamic changes in international students’ cross-cultural abilities in terms of areas, such as individual cultural awareness, social network support, learning methods, and communication in a new cultural environment. There were changes in terms of learning, communication connection, self-management, and cultural awareness, and the balance of social network, source country, and resource relationship between study places all play an important role. Meanwhile, international students are more likely to present themselves in terms of their university identity than in terms of their religious or ethnic identity. In addition, connectivity, as an important manifestation of cross-cultural competence, refers to the virtual or real relationships that international students maintain with people, communities, organizations, etc. It is influenced by various factors such as local social networks, belonging, culture, values, politics, media, the learning environment, and location. One participant also argued:

*I found that when international students’ cross-cultural adaptability is weak, our studies, psychology, and living conditions are affected. We feel lost on the Chinese campus and can become very depressed during the learning process. To help international students like me achieve rapid acculturation, higher education institutions should put forward peer-mentoring programs to promote international students’ cross-cultural adjustment.*

(HML)

It is suggested that it is more beneficial for overseas students to establish social networks with local students rather than students from their own country or other countries to promote their cross-cultural adaptation. In fact, the establishment of social networks is also a manifestation of the promotion of intercultural competence through more cross-cultural dialogs. In addition, some scholars have stressed the importance of educational services for overseas students, which should be promoted from various aspects, such as education, teaching, scientific research, teacher education, and student management, to enhance overseas students’ learning adaptability and cultural integration ability. One participant also commented:

*I think that universities should provide our international students with support in areas beyond academic studies, such as job-hunting skills and cultural intelligence, to help them find better jobs. At the same time, some students tend to lack cultural confidence when they want to stay in the country where they study to find a job, or when their major requires them to deal with local people, which will have an impact on their self-confidence. In general, cross-cultural competence affects all aspects of international students, and language, cultural identity, and cultural integration ability also affect cross-cultural competence. Therefore, the countries of origin of international students need to pay attention to the cultivation of students’ international and cross-cultural competence to provide pre-departure support for students studying abroad. At the same time, the destination country should also provide corresponding cross-cultural adaptation support after the student starts their life as an international student to better promote their studies, work, and life.*

(WYX)

In general, we should pay attention to the important role of international students in spreading China’s image and the important role of cultivating international students’ understanding of Chinese culture in spreading China’s image. In terms of content, cross-cultural adaptation is still the primary research issue in international academic circles. Current research mainly explores the cross-cultural adaptation and ability of international students, as well as the local impact of cross-cultural adaptation on the studies, work, and lives of international students. With the development of China’s economy, the number of Chinese overseas students is gradually increasing, and they make up a major part of the number of overseas students in the main destination countries. In addition, due to the vast differences between Chinese culture or Asian culture and Western culture, the cross-cultural problems of Chinese or Asian students have often become a hot topic for Western scholars to study. In addition, Asian, Hispanic, African, and other students from ethnic minority backgrounds are also vulnerable to discrimination in Western countries, and the psychological and acculturation problems brought by this are still attracting the attention of scholars.

## 4. Discussion

Along with the results discussed above, we found that in terms of curriculum and teaching, higher education institutions should emphasize the diversification of curriculum content, and local cultural characteristics, such as an excellent traditional culture or industrial culture, should be incorporated into the curriculum’s content depending on the school [42]. At the same time, universities and colleges should pay attention to the introduction of China’s basic political system and laws and regulations to reflect Chinese culture and core values, so that international students can understand many aspects of China. In teaching, teachers should actively adopt situational teaching methods, pay attention to the development of heuristic teaching, use local resources to carry out activities to gain experience, and establish a cultural experience base. In terms of management, against the background of considering the diversity of students, the convergent management of life and study should be strengthened [43].

The results of our analysis suggest that the language threshold for international students in programs taught in Chinese should be increased, for example, by making level 6 or level 8 of the Chinese Proficiency Test mandatory. Students who do not meet the language requirements must first participate in a preparatory Chinese program. It is recommended that the Chinese-level assessment of international students be strengthened in order to lay a solid language foundation for their subsequent participation in professional courses. In addition to the Chinese courses stipulated in the syllabus for international students, courses related to local dialects can be added as needed. The school can set up a Chinese club for international students and organize weekly off-campus practice activities, thus not only allowing international students to practice and improve their Chinese level but also letting them see more aspects of life in China.

In addition, we found that where teachers are concerned, more training opportunities in foreign languages, especially English, should be provided for teachers with science and engineering backgrounds—for example, the establishment of language service centers within schools to provide free language training courses to teachers who need them. In addition, it is suggested that teachers who have taken the IELTS or TOEFL tests and achieved the required scores in listening and speaking should be included in the selection criteria for teachers of programs taught in English. The relevant departments and colleges of universities should further promote exchanges and interactions between Chinese and foreign students, implement an “inclusive” policy, and open all associations and volunteer programs that are available to Chinese students to international students. Combining textbook content with real-life experience is the best way to enhance international students’ in-depth understanding and recognition of Chinese culture.

In addition, it was found that the training and assessment of educators of international students should focus on enhancing their cultural literacy and cross-cultural working abilities [44]. In the campus environment, we should reflect on the characteristics of the campus in terms of both its Chinese characteristics and multicultural characteristics and attempt to enhance its cultural adaptation and cultural identity. In terms of the self-governance of international students, we should pay attention to the platform-building of international students’ associations and encourage diversified activities. In terms of resource utilization, information channels should be unimpeded and expanded to promote international students’ use of local resources, such as psychological counseling and art activities [45].

For the academic aspects, the previous studies have also highlighted that, in the actual process of carrying out teaching and management activities, colleges and universities need to start from an international perspective and form new thinking about current management concepts and teaching modes. For example, Maeder-Qian (2018) suggested that colleges and universities need to establish the concept of international development to enrich and expand school curriculum resources. This should not be affected by traditional teaching ideas or by too much emphasis on imparting knowledge or technology. Educators should focus on cultivating students’ international vision and adaptability to the international community. Yan and Berliner (2011) also indicated that both colleges and universities can scientifically integrate cross-cultural handover theory into disciplines by improving their own education links and talent training strategies, as a result a teaching environment with more international adaptability can be gradually formed [46]. Xia and Duan (2020) also highlighted that the cross-cultural adaptation of overseas students comes from their own values and from the stimulation of the external environment. For example, the exclusivity generated by human beings is not conducive to the rapid narrowing of distances and emotions between people. Hu et al. (2019) argued that when international students cannot really integrate into campus life or class activities, it is bound to bring them great ideological trouble, resulting in great inadaptability. In particular, the differences between students from different countries due to their social or political cultures may lead to friction and disputes in the process of exchange. Students from different countries will have certain differences in terms of their outlooks on life, values, world outlooks, etc., which can give rise to problems in cross-cultural adaptation. As for the analysis of the cross-cultural adaptation of students from South Asia, religious beliefs are likely to become the key factor causing such problems.

This study has both theoretical and practical implications. For the theoretical implications, it provides an in-depth understanding and discussion of how international students in China should be encouraged to actively carry out contacts and activities within the local Chinese campus context, especially in the post-COVID-19 era. Regarding its practical value and implications, this study established that the cross-cultural understanding and global competency education of local Chinese students should also be actively carried out. The difficulty of adjusting to a Chinese cultural context for international students is due more to the psychological and behavioral resistance caused by the unknown and uncertain. Therefore, in future research on the diversity of international students in China, we should also consider the educational objectives of international students in China, promote their understanding of Chinese culture in an all-round way, and pay attention to the coexistence of rationality and sensibility and to the coexistence of heterogeneity and experience [47]. Promoting international students’ understanding of Chinese culture can play an important role in the development of international students’ education, bilateral economic cooperation, China’s soft power, and China’s international image communication. The promotion of an understanding of Chinese culture should not only start from the cultural aspects but should also build an education system that promotes Chinese culture in terms of all aspects [48,49,50].

This study has some limitations. For future studies, more diverse countries could be added to explore more holistic contexts regarding the cultural adaptation and mental health of international students [51]. For example, Glass. and Hou (2022) explored the intersections of identity and status in international students’ perceptions of culturally engaging campus environments. Sheng et al. (2022) also analyzed the impacts of academic adaptation on psychological and sociocultural adaptation among international students. In addition, a survey of international students could be designed to conduct a large-scale data analysis of international students’ cultural adaptation. China is not the only country with a foreign student population. For example, Antonio and Baek (2022) used the survey measures to figure out international male graduate students’ sense of belonging in electrical engineering. More comparisons among different countries could be added in future research [52]. In addition, more comparative studies could be added to examine the similarities and differences with other related literature studies on exploring the adjustment to different cultures and mental health issues among foreign students on various campuses [53]. For instance, Bhat et al. (2021) explored the experiences of alcohol among Asian international students in Australia.

## 5. Conclusions

In summary, the results of this study align with the previous studies to explore the adjustment to the culture and mental health issues among foreign students on Chinese campuses during the COVID-19 pandemic. In addition, new aspects have been introduced and discussed in this study. For example, we found that international students tend to have a limited understanding of Chinese culture and rely on very few channels for information—in particular, the Internet, teachers’ lectures, and daily life—which can easily result in mental health problems and thoughts of marginalization. Compared with the previous relevant studies of Maeder-Qian (2018), Xia and Duan (2020), and Yan and Berliner (2011), this study showed that international students’ mental health problems are subjectively positively correlated with their own personality, cultural intelligence, and cultural identification ability and objectively related to their cultural distance and all aspects of the educational work of international students. There are also some similarities and differences with other related literature studies, such as the research themes and research contextual backgrounds. The innovations of the study compared with other literature are to use a collaborative ethnography approach to explore the adjustment to Chinese culture and mental health issues among foreign students on Chinese campuses during the COVID-19 pandemic. Chinese higher education institutions are suggested to strengthen their attention to the mental health of international students in China and promote international students’ cross-cultural adaptation abilities and understanding of Chinese culture.

## Figures and Tables

**Table 1:** The participants’ basic information and descriptions.

No	Name	Major and Nationality	Class	Gender	Institution	No	Name	Major and Nationality	Class	Gender	Institution
1	WYY	Chinese/U.S.	16	Male	B University	43	ZYP	Accounting/U.S.	6	Male	B University
2	WQQ	Chinese/UK	16	Female	B University	44	SRR	Accounting/U.S.	6	Male	C University
3	ZQQ	Chinese/U.S.	16	Male	C University	45	ZYH	Painting/U.S.	4	Male	B University
4	LLY	Chinese/U.S.	16	Female	B University	46	ZCY	Painting/UK	4	Female	B University
5	WTT	Chinese/UK	16	Female	B University	47	SJJ	Painting/U.S.	4	Female	C University
6	CHB	Chinese/U.S.	16	Female	B University	48	ZJZ	Painting/U.S.	4	Male	B University
7	XN	Chinese/UK	16	Male	C University	49	ZYW	Painting/UK	4	Male	D University
8	YJH	Chinese/U.S.	16	Female	B University	50	WSR	Painting/UK	8	Female	B University
9	LQ	Chinese/UK	16	Male	B University	51	LX	Digital/UK	1	Female	D University
10	HML	English/UK	13	Female	C University	52	DJR	Digital/U.S.	1	Male	B University
11	JYF	English/UK	13	Male	D University	53	CY	Digital/UK	1	Male	A University
12	CYY	English/UK	1	Female	A University	54	PG	Digital/Korean	1	Female	A University
13	ZWY	English/UK	1	Male	B University	55	QCF	Digital/U.S.	1	Male	B University
14	WYP	English/UK	4	Female	A University	56	SJZ	Digital/Korean	1	Female	B University
15	WZX	Math/UK	2	Male	B University	57	ZH	Digital/U.S.	1	Male	E University
16	LMJ	Geography/Korean	1	Male	A University	58	ZWH	Digital/UK	1	Female	B University
17	XXY	Geography/Korean	1	Female	B University	59	YLN	Digital/Korean	1	Female	B University
18	SXR	Geography/New Zealand	1	Female	E University	60	ZXX	Digital/Korean	1	Female	A University
19	LJJ	Geography/New Zealand	1	Male	B University	61	ZLJ	Digital/U.S.	1	Female	B University
20	WD	Geography/Korean	1	Female	B University	62	FJ	Digital/Korean	1	Female	A University
21	SJL	Music/Korean	4	Female	F University	63	HQQ	Digital/Korean	1	Female	F University
22	ZZQ	Music/U.S.	4	Male	B University	64	ZHD	Digital/U.S.	1	Female	B University
23	LWY	Music/Korean	4	Female	F University	65	ZHX	Digital/Korean	1	Female	F University
24	LJQ	Music/U.S.	5	Male	B University	66	ZYY	Digital/U.S.	1	Male	B University
25	CY	Music/Korean	5	Female	B University	67	TZY	Digital medium/New Zealand	1	Female	F University
26	LX	Music/Korean	5	Male	F University	68	HHL	Digital/U.S.	2	Female	B University
27	LSF	Music/U.S.	5	Female	B University	69	SFD	Digital/Korean	2	Female	C University
28	ZMH	Music/Korean	5	Female	B University	70	LQX	Digital/Korean	2	Female	B University
29	WY	Music/Korean	5	Female	F University	71	LYP	Digital/Japan	2	Male	B University
30	WWZ	Music/Japan	6	Female	A University	72	SRR	Digital/Japan	2	Female	B University
31	DYZ	Music/Japan	6	Female	B University	73	XWM	Digital/Japan	2	Female	B University
32	ZYT	Music/Japan	6	Female	A University	74	HXD	Digital/Canada	2	Female	B University
33	SZB	Accounting/Japan	1	Female	B University	75	DMM	Digital/Canada	2	Female	B University
34	ZYH	Accounting/Canada	1	Female	B University	76	LXJ	Psychology/Japan	2	Female	C University
35	ZR	Accounting/Japan	1	Female	B University	77	ZMH	Psychology/Canada	2	Female	B University
36	XLY	Accounting/Japan	1	Female	A University	78	ZGR	Psychology/Canada	2	Female	F University
37	DYY	Accounting/Canada	1	Female	B University	79	ZQY	Education/Canada	4	Female	B University
38	JJJ	Accounting/Canada	1	Female	B University	80	WXL	Education/New Zealand	4	Female	C University
39	LJY	Accounting/Canada	1	Female	B University	81	DHY	Education/New Zealand	4	Female	B University
40	ZXL	Accounting/New Zealand	6	Female	A University	82	LYY	Education/New Zealand	4	Female	B University
41	ZY	Accounting/New Zealand	6	Female	B University						
42	JYT	Accounting/New Zealand	6	Female	F University						

**Note: **Classes 1 to 16 represent the administrative classes of the different international students, not their majors. In Chinese university management, it is customary to divide students into different administrative classes according to the number of students, and each class has a head teacher who is responsible for the management of their daily studies, lives, and work. The letters before the universities denote a particular university; that is, universities with the same letter sign mean that the participants in the research came from the same university as other students with the same letter sign.

**Table 2:** Higher-frequency words.

Words	Frequency	Weighted Percentage (%)
Cultural autism	19	1.72
Cultural adaption	15	1.36
Chinese cultural understanding	14	1.27
Cross-cultural adaptability	8	0.72
International students’ cultural difference	7	0.63
Social networks with local students	5	0.45
Chinese culture	3	0.27
International education	3	0.27
Internationalization	3	0.27
International students	3	0.27

## Data Availability

The datasets generated during and/or analyzed during the current study are available from the corresponding author upon reasonable request.
